# TestIME: an application for evaluating the efficiency of Chinese input method engines in electronic medical record entry task

**DOI:** 10.1186/s12911-019-0932-7

**Published:** 2019-12-05

**Authors:** Feihong Yang, Haihong Guo, Jiao Li

**Affiliations:** Institute of Medical Information and Library, Chinese Academy of Medical Sciences / Peking Union Medical College, Beijing, China

**Keywords:** Input method engine, Electric medical record, Text entry, Evaluation tool, Chinese

## Abstract

**Background:**

With the wide application of Electronic Medical Record (EMR) systems, it has become a daily work for doctors using keyboards to input clinical information into the EMR system. Chinese Input Method Engine (IME) is essential for doctors to convert pinyin to Chinese characters, and an efficient IME would improve doctors’ healthcare work. We developed a tool (called TestIME) to evaluating the efficiency of the current IMEs used in doctors’ working scenario.

The proposed TestIME consists of four major function modules: 1) Test tasks assignment, to ensure that participants using different IMEs to complete the same test task in a random order; 2) IME automatic switching, to automatically switch the input method engines without changing the experimental settings; 3) participants’ behavior monitoring, to record the participants’ keystrokes and timestamp during the typing process; 4) questionnaire, to collect the participants’ subjective data. In addition, we designed a preliminary experiment to demonstrate the usability of TestIME. We selected three sentences from EMR corpus and news corpus as test texts respectively, and recruited four participants in a medical school to complete text entry tasks using the TestIME.

**Results:**

Our TestIME was able to generate 72 files that record the detailed participants’ keyboard behavior while transcribing test texts, and 4 questionnaires that reflect participants’ psychological states. These profiles can be downloaded in a structured format (CSV) from the TestIME for further analysis.

**Conclusions:**

We developed a tool (TestIME) to evaluate Chinese input methods in the EMR entry tasks. In the given text input scenario in healthcare, the TestIME is capable to record doctors’ keyboard behavior, frequently used Chinese terms, IME usability feedback etc. These user profiles are important to improve current IME tools for doctors and further improve healthcare service.

## Background

With the wide application of Electronic Medical Record (EMR) systems, it has become a daily work for doctors using keyboards to input clinical information into the EHR system. According to the national health commission of China, the number of people discharged from public hospitals from January to November 2018 is 14.68 million [1], which means that doctors wrote the similar amount of medical records for patients’ visits.

Doctors use pinyin input method to type medical texts into EMR systems. A Chinese input method engines (IMEs) convert the pinyin to Chinese characters, words or phrases in the backend. Pinyin is an official phonetic coding system that uses Latin alphabet to represent sounds in standard Chinese, i.e. Putonghua [2]. Most IMEs are based on the rules of pinyin coding Chinese characters to realize the mapping from pinyin to Chinese characters.

Current IMEs cannot meet the doctors’ paces in their busy healthcare practices. Pan Junfei and Yao Xiang [3] have a study to improve the EMR entry efficiency of doctors based on functions of one pinyin input method. Actually, there is more than one mainstream IMEs, such as Sogou Pinyin [4], Baidu IME [5] and so on. Sogou also launched the “Sogou Input Method Doctor’s Version” [6] in September 2018, claiming to have collected 100,000 medical terms and design specifically for doctors.

In general text entry, the efficiency of input method engines have been improved greatly after years study [7–9]. For the special sentences structure and medical nomenclature in the EMR text, is the input methods having the same performance in the EMR entry task? To the best we know, this question is still unclear. In order to explore the efficiency of Chinese input methods in the EMR entry task and find approaches to design an IME for doctors, we designed a application named TestIME (the abbreviation of testing input method engines.)

In terms of evaluating the efficiency of IMEs, there are both theoretical framework studies and specific experiment studies under different scenarios. Soukoreff RW and MacKenzie IS [10, 11] proposed a framework for error analysis in text entry. Their framework combines the analysis of the presented text, input stream, and transcribed text. The analysis metrics include a unified total error rate, combining two constituent error rates: the corrected error rate (errors committed but corrected) and the not corrected error rate (errors left in the transcribed text). Both of them are based on keystrokes metric.

KySS [12] is a novel evaluation framework for IME proposed by effectively modeling user behavior during Chinese input processes. This evaluation framework aims to fast and accurately evaluate various IMEs from the perspective of user experience. It also uses keystrokes as core metrics.

To evaluate the usability of different input methods on mobile devices for historical African languages, Olaleye S and Suleman H [13] developed four different input methods (Xwerty, T9, Pinyin script and Hierarchical) and the text entry evaluation prototype (named Xamobile) using Java with Eclipse IDE, Android ADT and the Android SDK.

Qiu Liquan et al. [14] developed an open source testing tool for evaluating handwriting input methods, which is composed of two parts: a PC and a mobile client. The PC includes an automated test tool for Android device applications. The mobile client is designed and implemented based on the Android system. They conduct automatic testing on six leading Chinese handwriting IMEs (Baidu, Sogou etc.) to objectively evaluate and compare their recognition performance.

To evaluate “upper-bound performance” of two state-of-the-art mobile text input methods, speech recognition and typing on a touch-based keyboard in both English and Mandarin Chinese, Ruan Sherry et al. [15] developed a custom experiment test-bed app using Swift 2 and Xcode 7, and connected this app to a speech recognition system. The test-bed app presents phrases for transcription using both keyboard and speech as text input user interfaces, and all the operation information of the subject in each interface, such as the insertion and deletion of text and the corresponding timestamp, could be recorded for further analysis.

In this study, we developed TestIME (Testing Input Method Engines), which is able to record the behavior of participants in EMR text entry tasks, so as to improving the efficiency of IMEs. TestIME includes four major function modules: test tasks assignment, IME automatic switching, participants’ behavior monitoring, and questionnaire.

## Implementation

### Basic tools

Most public computers in Chinese hospitals run Windows operating system. Doctors are adept at using apps on the Windows platform to do routine medical works. To ensure that our tool is most closely to the actual work of doctors, TestIME needs to be designed as a platform compatible with Windows 7 and later. Fortunately, Microsoft provides a number of guiding frameworks for designing and implementing compatible platforms. In combination with practical needs, we decide to use Visual Studio 2019 Community [16] to create *TestIME* solution project, Windows Presentation Foundation (WPF) [17] to build the user interface (UI), and C# to develop program scripts.

Wen’juanxing [18], an online questionnaire production and distribution platform widely used in China, is characterized by simple production and exquisite interface. It can be used as a one-stop station to make the questionnaire and generate the url link. Then, we can integrate the link into the *WebBrower* component of WPF and write the corresponding function to listen to Window events. By contrast, redeveloping the questionnaire system using WPF is time-consuming and laborious.

In addition, most of IMEs are developed based on Microsoft’s Text Services Framework (TSF) [19], so TSF API is needed to interact with IME. TSF is a COM architecture (a set of interface design specifications advocated by Microsoft) implemented in C++, and C# cannot use directly. The open source library TSF-TypeLib [20] encapsulates the TSF interface to make it usable in C#.

Based on these basic tools, we implemented four major function modules: test tasks assignment, IME automatic switching, participants’ behavior monitoring, and questionnaire.

### Test tasks assignment

Participants were asked to transcribe the same test text multiple times using different IMEs. In order to avoid the situation that participants transcribe the same text continuously and the bias caused by growing familiarity with the texts, we developed a random test task assignment algorithm, which can be described as following:

1. Import the predefined CSV file which records test texts row by row. Initialize the list which contains the IMEs’ information.
2. Push the test texts in a stack data structure randomly. Combine the stack with single IME, which means the IME will be used by the participants to transcribe the random order test texts of the stack. And then push the combined structure in the global test task stack structure in a random order.
3. Pop the combine structure from the global test task stack one by one when participants transcribing.

### IME automatic switching

Using TSF-TypeLib, we implemented a *TSFWapper* class, which contains *GetName* and *SwitchIME* methods. The first method is used to get the information of current IME and the last one is to activate IME we specified. When test task popped, TestIME use the *SwitchIME* to activate the IME predefined in the combine structure mentioned above.

In addition, if participants pressed Ctrl+Shift key to switch IME they prefer, which is not allowed to do so in TestIME. Then, we need to listen to the keyboard events of the participants, and if the Ctrl+Shift key combination are pressed, we use *SwitchIME* to prevent the participants from changing the designated IME. So that participants can only use the specified IME to finish transcribing the test texts.

### Participants’ behavior monitoring

As mentioned in the relative works section, keystrokes metric is widely used in the evaluation of IME. When participants transcribing, they may commit errors and make corrections.

Consider the below example:

- **Presented Text:** 患者中年男性.
- **Input Stream:** 患者是 ← 中年男性.
- **Transcribed Text:** 患者中年男性.

The participant entered an incorrect Chinese character(“是”) that was deleted with a backspace(“←”). These keystrokes do not appear in the transcribed text, hence the transcribed text is error free. According to the text entry analysis framework [10, 11], we need record the “Input Stream” when transcribing and save its data as a CSV file format for ease of analysis. The main fields information for the CSV file is shown in Table 1.

**Table 1:** Fields of CSV File

Name	Description
KeyBoard	The keystrokes when participants transcribing. Its value is pinyin sequence separated by’. For example HUAN’Z.
KeyLength	The Length of “KeyBoard” field. For example, the length of HUAN’Z is 5
MapText	Chinese characters converted from the “KeyBoard” field. For example, the “MapText” of HUAN’ Z is Chinese character, “患者”.
TimeStamp	Time point at every keystroke typed.
CurText	Chinese input stream in the current timestamp.

To record the participants’ keystrokes mentioned above in the backend, we developed a keyboard hook, which is inspired from the implementation of Dylan’s open source codes [21] and can be used to globally monitor the keyboard events to get the keystrokes typed by the participants. And a text difference detector, whose code is written in reference to the open source library Diff-Match-Patch [22], have also been implemented to get the transcribed text’s changing after every valid keyboard event.

### Questionnaire system

In addition to the objective metrics recorded by TestIME in backend, the subjective evaluation about corresponding IME and the feedback of participants are also important for our study, which can be helpful when we analysis the different results of participants in the same test task. Since using C# rebuild a questionnaire system is time-consuming and a third-party tool (mentioned in the Basic Tools subsection) named Wen’juanxing can meet our requirement, we have developed a *WebBrower* window to load the url generated by it. Then the making and collecting of questionnaire can be finished in the third-party platform.

In Wen’juanxing, we have designed two type of questionnaires. One of them is for collecting participants’ base information about demographic characteristics, experience of EMR entry, psychological state when participating in the experiment. Another is mainly used to survey participants’ work load after every text entry task finished. This paper use NASA-TLX (Task Load Index) [23, 24] to evaluate participants’ text entry task load, PHQ-9 [25] for participants’ depression diagnostic and severity measure, GAD-7 [26] to assess generalized anxiety disorder.

### Workflow and user interface

**Fig. 1:**
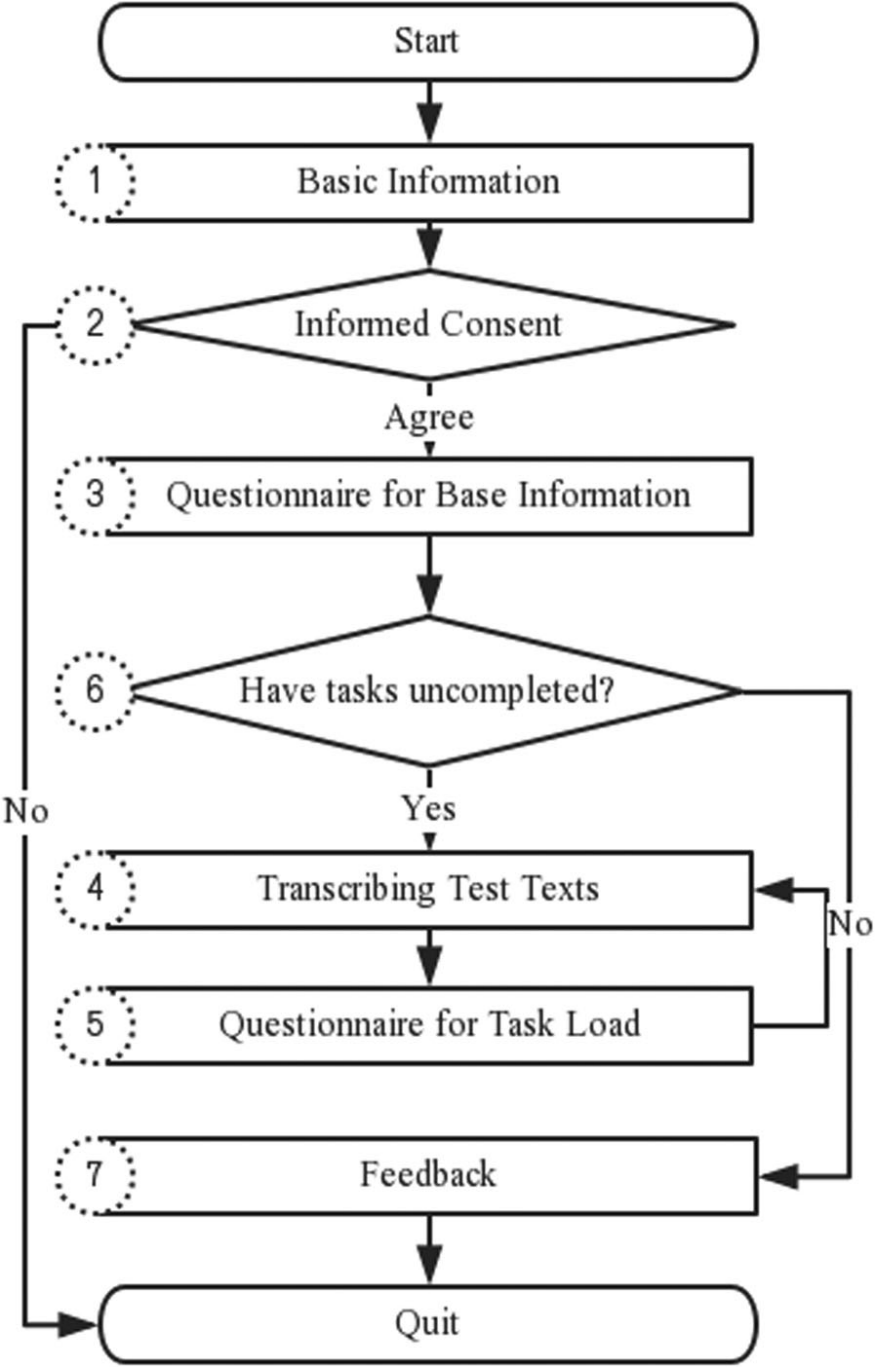
TestIME Workflow

**Fig. 2:**
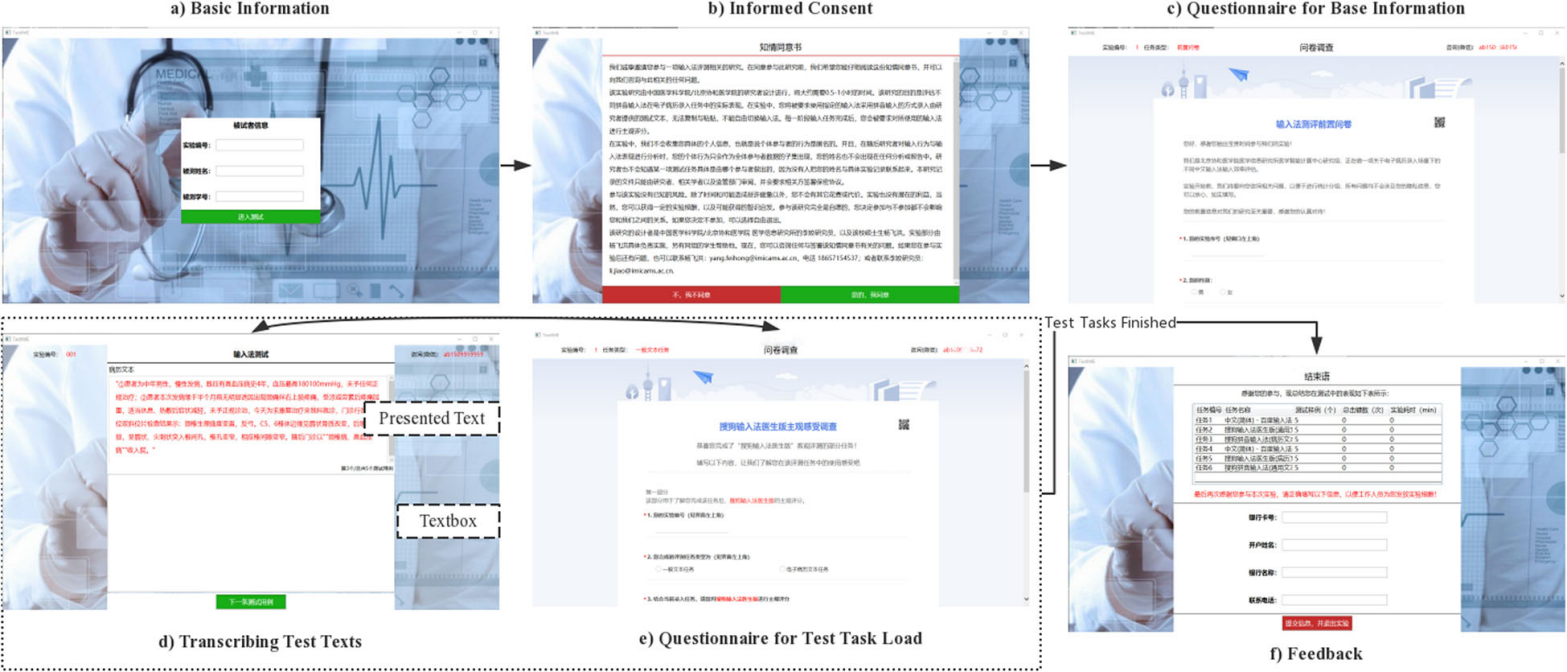
TestIME UI. Arrows indicate the order in which software Windows switch. For example, the arrow in the window “Basic Information” points to “Inform Consent”, indicating that the participant will go from “Basic Information” to “Inform Consent”

As shown in Fig. 1, TestIME work flow include 7 steps, the details and the corresponding user interfaces (UI) are as following:

- **Step 1** Basic Information (Fig. 2a). Participant is asked to fill in basic information, such as their ID, name and so on. Meanwhile, TestIME initializes the experimental configuration in the backend, such as importing presented texts and assignment test tasks in a random order.
- **Step 2** Informed Consent (Fig. 2b). Inform participant that what is to be observed and recorded in the experiment. Participant can make a choice to join in the experiment, or refuse and then quit the experiment.
- **Step 3** Questionnaire for base information (Fig. 2c). If participant chooses to join the experiment in Step 2, he/she is required to fill in a questionnaire about his/her demographic characteristics, experience of EMR entry, and psychological state when participating in the experiment.
- **Step 4** Transcribing Test Texts (Fig. 2d). Participant is required to use the specified IME to transcribe the presented text. The time is not limited, but he/she is not allowed to switch the IME and the copy-paste is also banned.
- **Step 5** Questionnaire for task load (Fig. 2e). Collect participant’s subjective scores about the corresponding IME in Step 4, and the text entry task load.
- **Step 6** Test Tasks unfinished? Repeat steps 4 and 5 until all test tasks have been completed.
- **Step 7** Feedback (Fig. 2f). Give feedback to participants about their general performance, such as the time consumed, the number of tasks completed and so on.

### Preliminary experiment settings

**Fig. 3:**
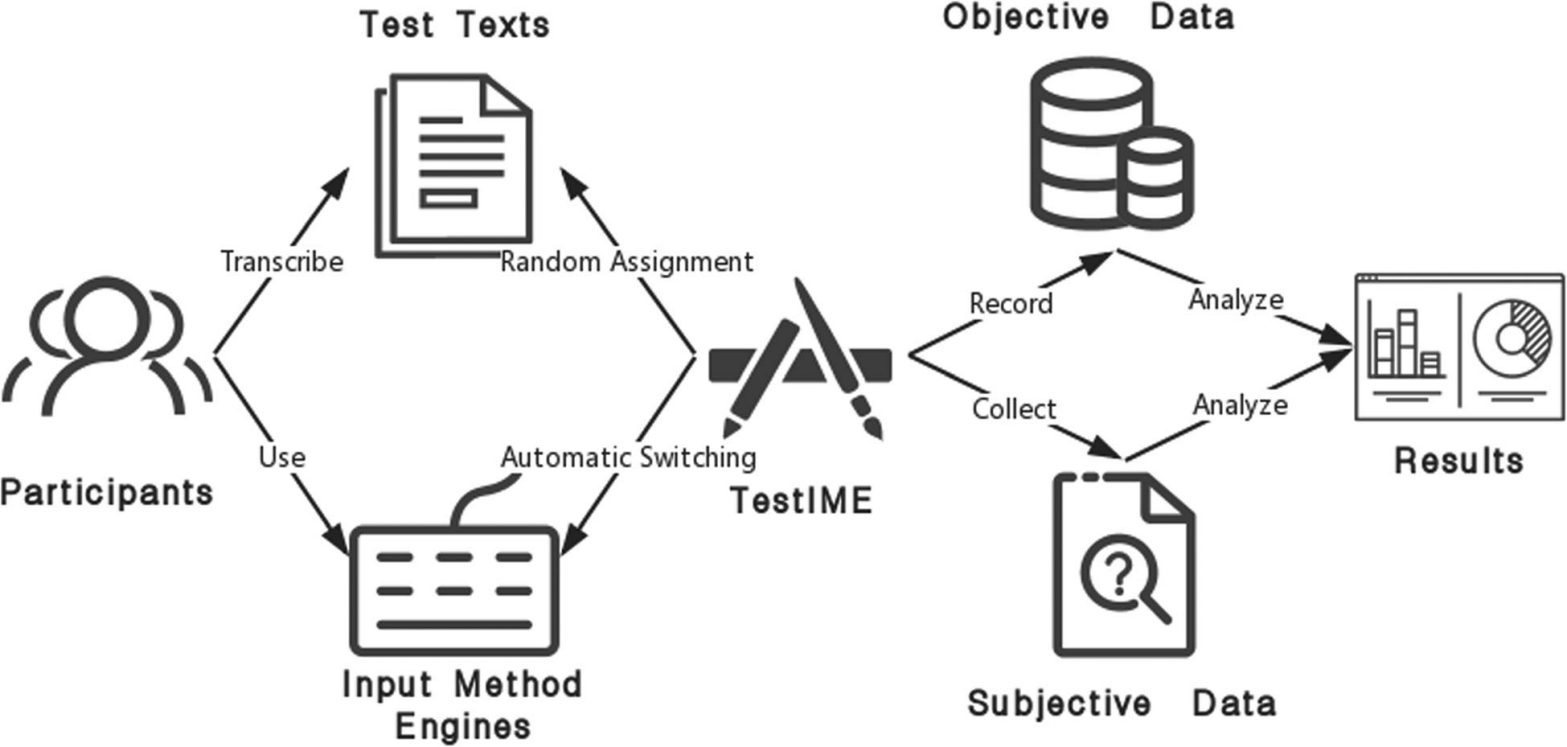
The Experiment Work Flow

To explain how TestIME works and what TestIME does in investigating the current input method engines, we designed a preliminary experiment. As shown in Fig. 3, the proposed TestIME is just one part of the whole experiment. What we need to do is to recruit participants, select input method engines and test texts for evaluating. And then TestIME is used to assign test tasks, monitor participants’ behaviors and collect data. At last, we need make an analysis about the data.

#### Participants

We invited four participants, who come from different majors of Peking Union Medical College. They were numbered as “001”, “002”, “003” and “004”. All of them agree to join our experiment after reading our Inform Consent. In a quiet office, participants were asked to use different Chinese input method engines to transcribe the presented texts in TestIME.

#### Input method engines

Three input methods engines were selected in our experiment, which are named as Sogou Pinyin, Baidu Input Method, Sogou Input Method Doctor’s Version. The first two IMEs are most popular used by Chinese doctors in EMR entry task. To avoid the unintentional effects on the IME providers, the specific name of the IME should not be mentioned in the experiment. So, we named the three IMEs as IME1, IME2 and IME3 respectively.

Table 2 gives some relevant information about the three IMEs.

**Table 2:** Information of three IMEs

	IME1	IME2	IME3
Version	9.3	5.5	1.1
Package Size	42.2 MB	43.2 MB	48.9 MB
Release Date	2019-04-11	2019-04-19	2018-09-19

#### Test texts

Three EMR sentences are randomly selected from the open source EMR datasets released by China Conference on Knowledge Graph and Semantic Computing [27]. To ensure the diversity of the test corpus, three EMR sentences with different length are selected from different text contexts. In addition, we choose three news sentences from reported by People Daily in 2014 [28]. The news sentences are baseline data which are selected in the same way as we did for EMR sentences.

**Fig. 4:**
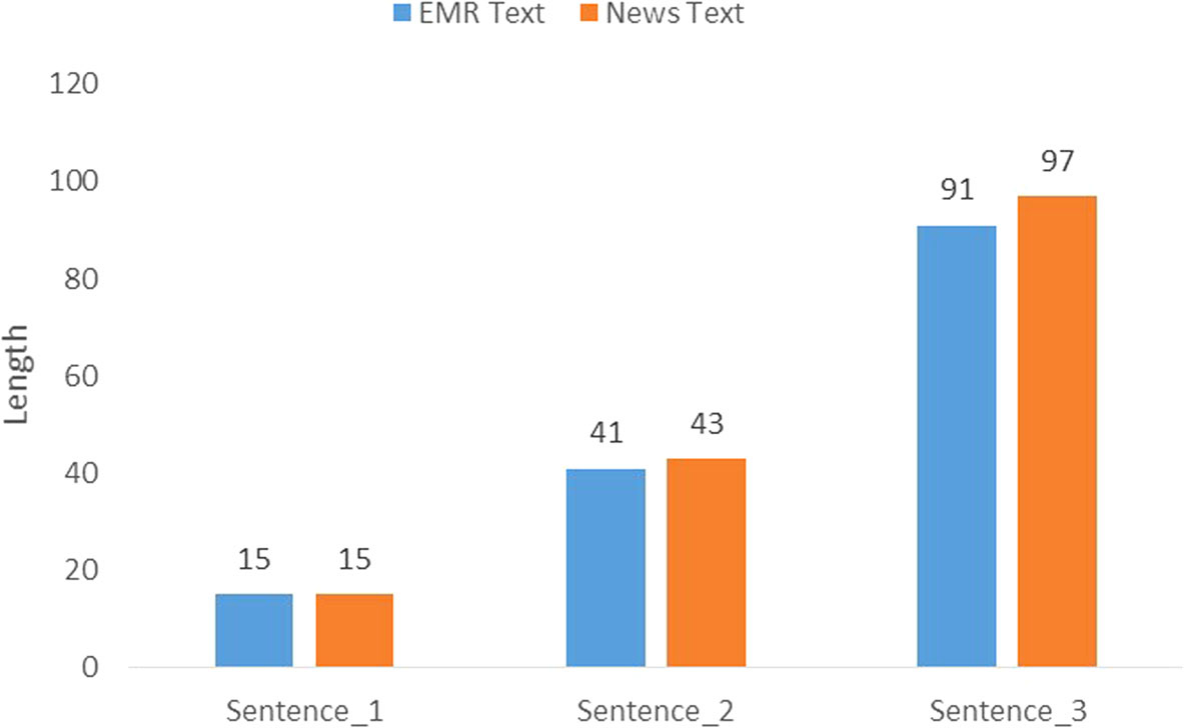
The Length of Test Sentences

In order to shorten the experimental duration of participants and reduce experimental errors caused by fatigue, length of the three EMR sentences is limited in 15, 41 and 91 respectively. For comparability, the length of the news sentence corresponds to that of the EMR sentences. The length of the six testing sentences are shown in Fig. 4.

We numbered the test tasks as shown in Table 3. “IME1 + News Text” means that participants use IME1 to transcribe the three news text, and other expressions are similar.

**Table 3:** Task ID And Name

Task ID	Task Name
1	IME1 + News Text
2	IME1 + EMR Text
3	IME2 + News Text
4	IME2 + EMR Text
5	IME3 + News Text
6	IME3 + EMR Text

#### Experiment configure

To ensure that the proposed method for evaluation is fair and reasonable, we make sure that all the participants completed text entry tasks in the same experiment configure. In this experiment, TestIME run on a Windows 10 PC with Intel(R) Core (TM)i7-8550 U CPU @18.0GHZ 2GHZ, 8GB RAM. For the online- learning capability of input method engines may cause bias, we refresh the experiment configure every time when one participant finish the test tasks. In a same rule, three IMEs are installed before the experiment or uninstall after.

## Results

Four participants participated fully in our experiment. During the experiment, TestIME ran stably, the page loaded smoothly, and no abnormal conditions were reported. The random assignment tasks of the participants are shown in Table 4. Each participant’s test tasks order is automatically generated by the function module “Test tasks assignment”, and the automatic switching of IME is realized by using the function module “IME automatic switching”.

**Table 4:** Participant’s test tasks order

Participants ID	Test Tasks ID					
001	4	6	1	3	5	2
002	4	6	1	3	5	2
003	4	6	1	3	5	2
004	2	5	4	1	6	5

### Objective data

**Fig. 5:**
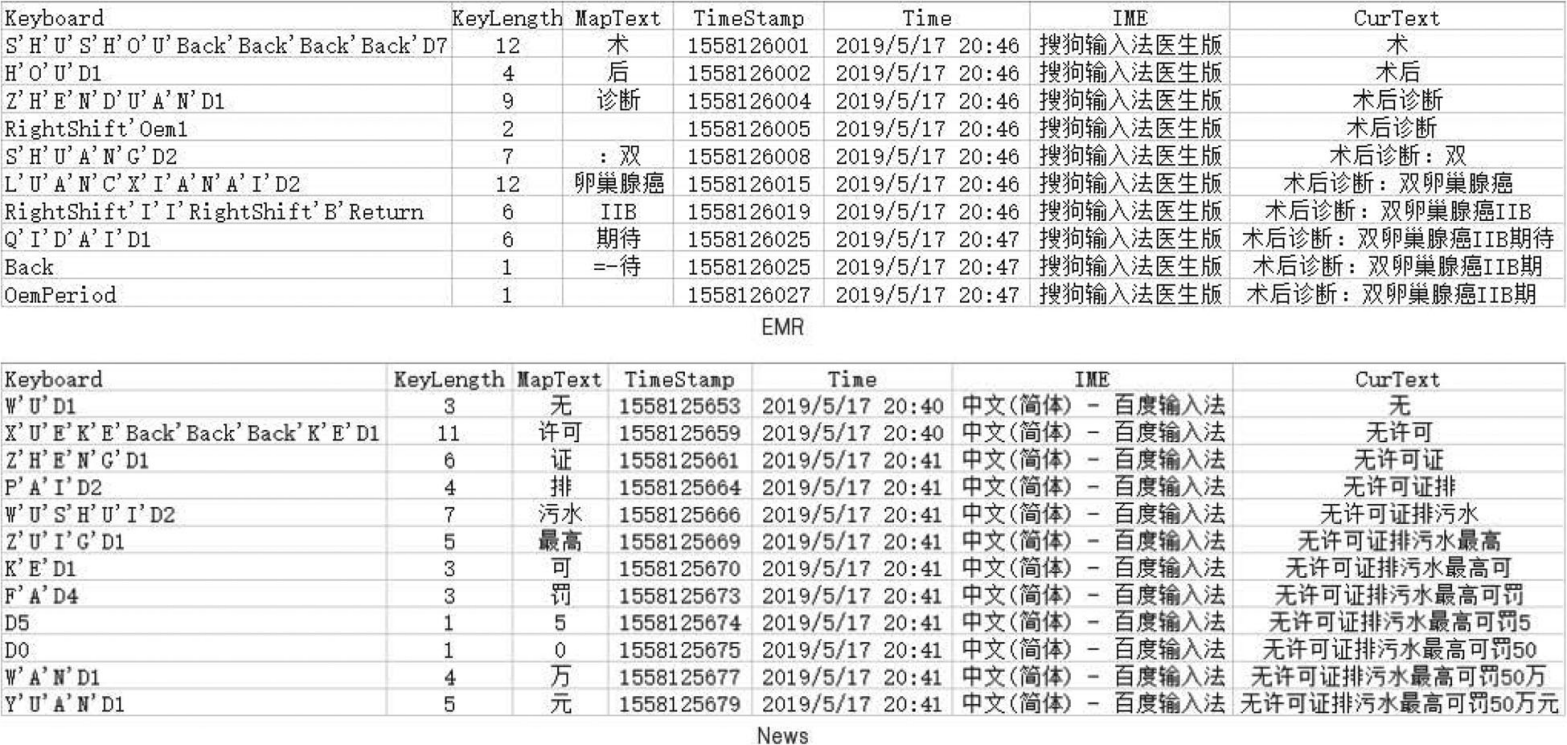
Tow CSV files generated by TestIME

The preliminary experimental results showed that TestIME worked well as expected. TestIME logged each of the transcribed sentence as one csv file, resulting in 6 csv logging files for each of the three IMEs per participants. Each csv file was named as a format, such as “001_1558125681.csv”, and contains the following 5 fields: KeyBoard, KeyLength, MapText, TimeStamp, and CurText. The description of these fields are listed in Table 1. Thus we have recorded 72 csv files for the four participants. Two of them are shown as examples in Fig. 5.

**Fig. 6:**
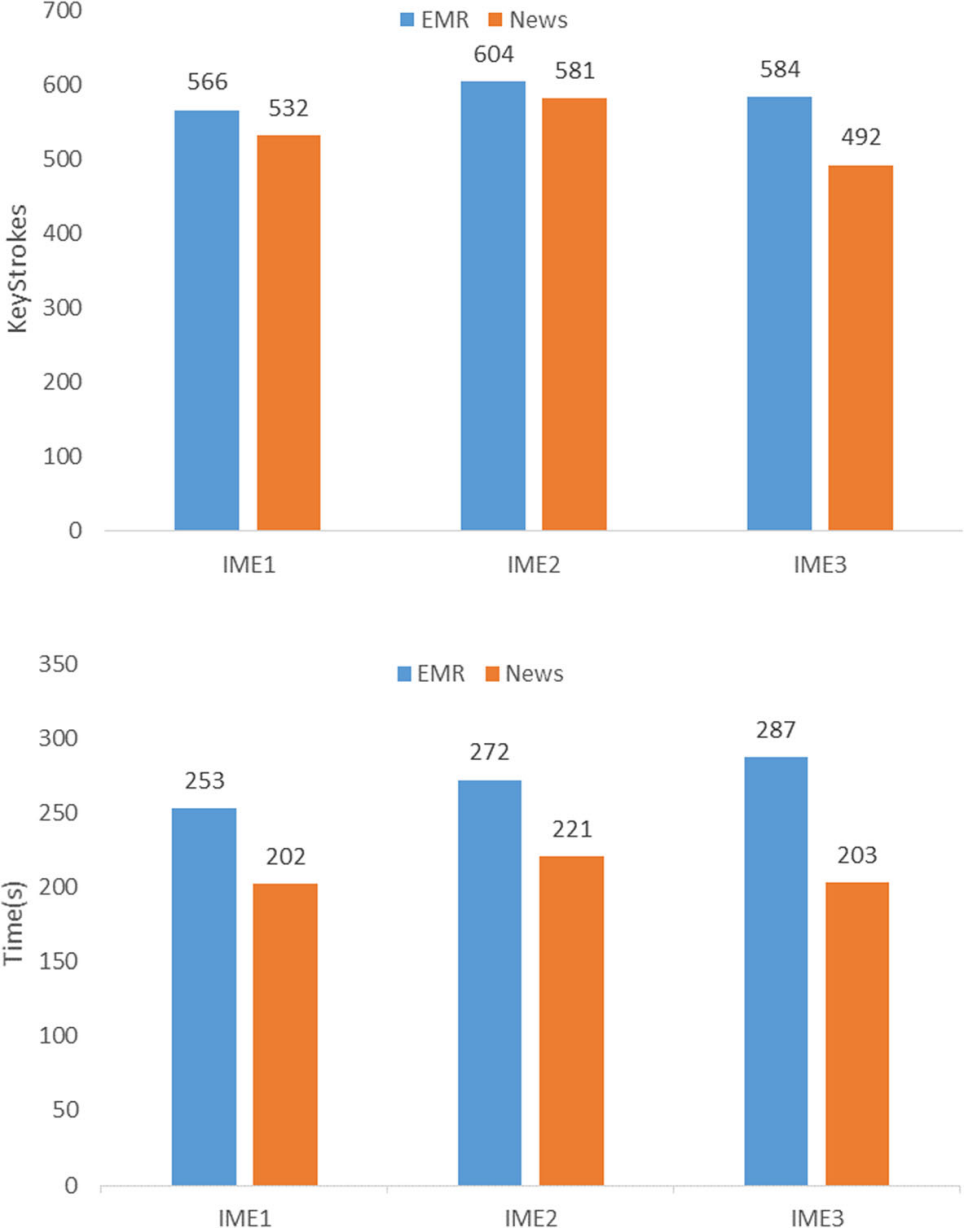
Keystrokes and Time consuming of IMEs

Based on these data, we calculated the average number of keystrokes and the average time taken by the four participants in test each task. When participants used IME1 to transcribe three news sentences, they had an average of 532 keystrokes and an average of 202 s time taken. By contrast, when participants used IME1 to transcribe three EMR sentences, the average number of keystrokes and the average time taken increased by 34 and 51 s, respectively. Similar trends were observed in EMR sentences and news sentence transcription tasks using two other input methods. The details are shown in Fig. 6.

### Subjective data

**Fig. 7:**
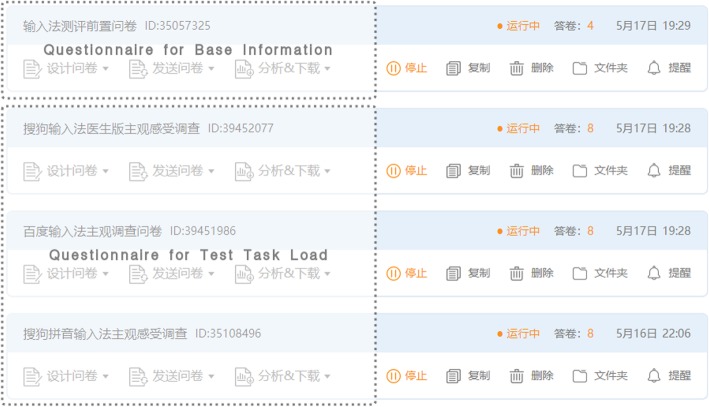
Questionnaires collected by TestIME

As we explain in section *Questionnaire System*, Participants’ subjective metrics mainly come from NASA- TLX (Task Load Index), PHQ-9 and GAD-7. The experimental results showed that TestIME worked as expected, we collected 4 questionnaires for participants’ base information and 24 questionnaires for test task load. Figure 7 is the dashboard of our experiment on the Wen’juanxing.

According to the questionnaire information, we obtained participants’ anxiety and depression scores as shown in Table 5.

**Table 5:** Participants’ PHQ-9 and GAD-7 Scores

Participants ID	PHD-9	GAD-7
001	2	1
002	5	6
003	5	4
004	1	0

**Fig. 8:**
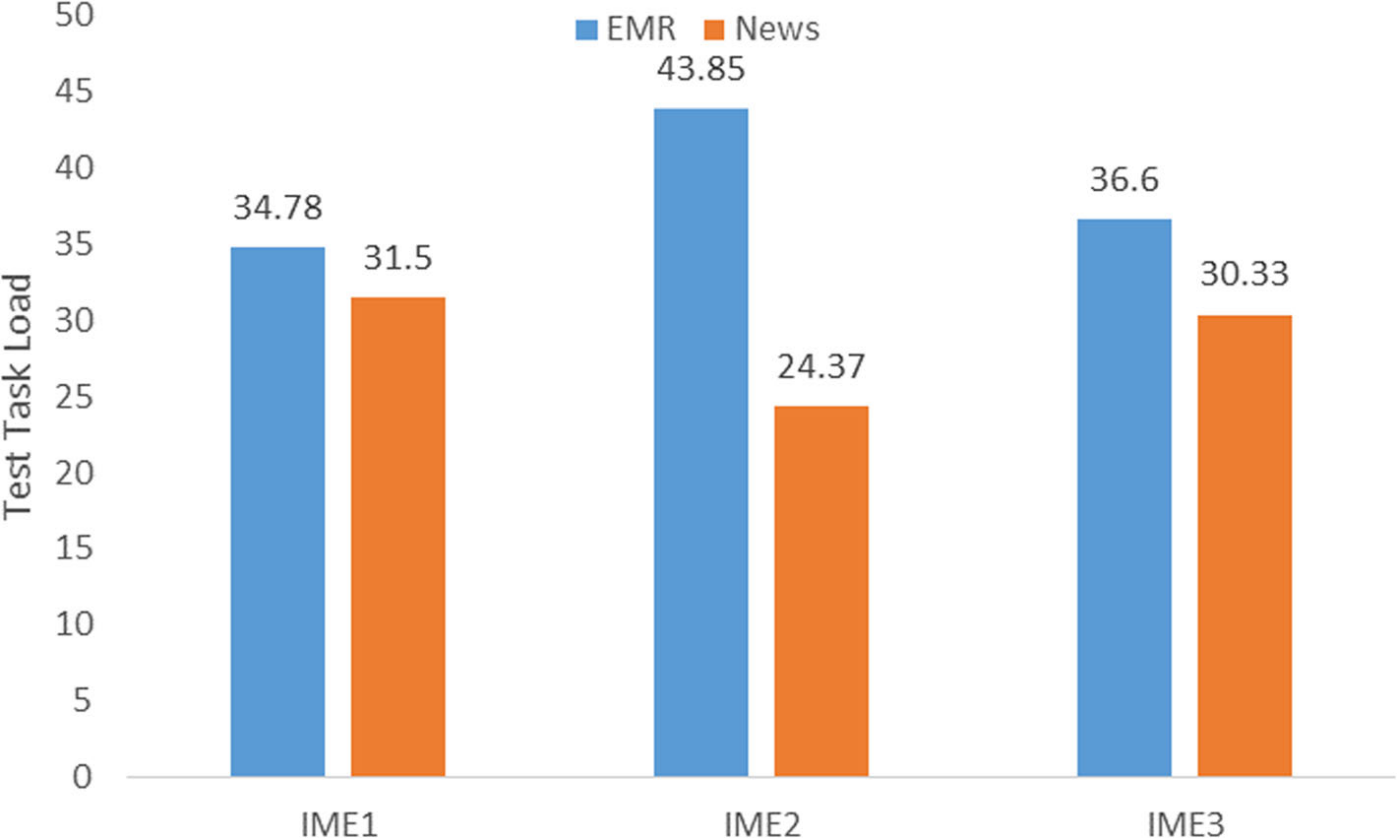
The participants’ test task load

We calculated the average task load score of participants who transcribed the assigned six sentences using the three IMEs. When participants using IME1 to transcribe three news sentences, their test task load score averaged 31.5. By contrast, the average task load score increased by 10.4% after three EMR sentences were copied using IME1. Participants who copied six sentences using the other two IMEs showed a similar upward trend. The details are shown in Fig. 8.

## Discussion

In this section, we focus on the internal reasons for obtaining the test results data and the features that TestIME has compared to existing tools.

### Test results data

From the preliminary experimental results, we found that when the assigned three IMEs were used by participants to transcribe EMR sentences, they all performed poorly according to the objective indicators of average keystroke times and average time spent, and the subjective indicators of average test task load score. It is surprisingly that the Sogou Input Method Doctor’s Version with the help of medical dictionary does not outperformed the other two general IMEs, and the reasons need to be further studied. The reasons for the poor performance of the selected three IMEs may be as following:

1. The performance of IMEs in EMR entry task is poor, so it is necessary to seek for improving the input efficiency.
2. The test corpus or the number of participants in the preliminary experiment may be too small to make subjective conclusion about the tested IMEs. Therefore, subsequent work should increase the number of participants and test corpus.
3. The three IMEs selected in the preliminary experiment are not all the input methods commonly used by participants, and the input habits of participants may cause experimental errors. In the future work, we will expand the scope of IMEs to be tested through a sound marketing and literature review.

### Compare to existing tools

Compared with other IME evaluation tools, the proposed TestIME is special and innovative in the following three aspects.

1. TestIME runs on Windows 7 and later system platforms, which is the main system used by doctors in office. While, most of the other evaluation IME tools run on IOS or Android mobile platforms.
2. TestIME is designed specific for the scene of EMR entry. With the popularization and application of EMR systems, the entry of EMR has become one of doctors’ daily work, and IMEs will affect their clinical work efficiency. Comparing the efficiency of IMEs in the scene of EMR entry is of great significance for studying how to improve IMEs. Although other existing evaluation software can also achieve this scenario design by selecting EMR corpus, most of them run on mobile platforms, which is quite different from the real world doctor working scenario.
3. TestIME can not only record the widely used keystrokes-based text entry indicators, but also collect subjective information of participants during the experiment, such as test task load and PHQ-9.

## Conclusions

We developed a tool (TestIME) to evaluate Chinese input methods in the EMR entry tasks. In the given text input scenario in healthcare, the TestIME is capable to record doctors’ keyboard behavior, frequently used Chinese terms, IME usability feedback etc. These user profiles are important to improve current IME tools for doctors and further improve healthcare service.

In future, we would apply the TestIME to assess the Chinese input tools used by doctors in the EMR typing scenarios such as inpatient diagnostic report and outpatient family member history entry tasks.

## Availability and requirements

**Project name:** TestIME.

**Project home page**: 10.5281/zenodo.3239337

**Operating system(s)**: Windows 7 and later.

**Programming language:** C#.

**Other requirements:** TSF-TypeLib.

**License:** MIT License.

## Data Availability

The datasets and software supporting the results of this article are available in the trueto/TestIME repository, 10.5281/zenodo.3239337.

## Abbreviations

CSV: Comma-Separated Values
EMR: Electronic Medical Records
IME: Input Method Engine
TestIME: means that test input method engines
